# Raltegravir does not revert efflux activity of MDR1-P-glycoprotein in human MDR cells

**DOI:** 10.1186/2050-6511-14-47

**Published:** 2013-09-20

**Authors:** Maria Luisa Dupuis, Alessandro Ascione, Lucia Palmisano, Stefano Vella, Maurizio Cianfriglia

**Affiliations:** 1 Department of Therapeutic Research and Medicines Evaluation, Istituto Superiore di Sanità, Viale Regina Elena 299, Rome, Italy

**Keywords:** Raltegravir, MDR1-Pgp, Drug substrate, MDR1-Pgp induction, Antiretroviral treatment

## Abstract

**Background:**

Raltegravir (Isentress®)(RALT) has demonstrated excellent efficacy in both treatment-experienced and naïve patients with HIV-1 infection, and is the first strand transfer integrase inhibitor to be approved for use in HIV infected adults worldwide. Since the *in vivo* efficacy of this class of antiviral drugs depends on their access to intracellular sites where HIV-1 replicates, we analyzed the biological effects induced by RALT on human MDR cell systems expressing multidrug transporter MDR1-P-glycoprotein (MDR1-Pgp).

**Methods:**

Our study about RALT was performed by using a set of consolidated methodologies suitable for evaluating the MDR1-Pgp substrate nature of chemical and biological agents, namely: i) assay of drug efflux function; ii) analysis of MDR reversing capability by using cell proliferation assays; iii) monoclonal antibody UIC2 (mAb) shift test, as a sensitive assay to analyze conformational transition associated with MDR1-Pgp function; and iv) induction of MDR1-Pgp expression in MDR cell variant subjected to RALT exposure.

**Results:**

Functional assays demonstrated that the presence of RALT does not remarkably interfere with the efflux mechanism of CEM-VBL100 and HL60 MDR cells. Accordingly, cell proliferation assays clearly indicated that RALT does not revert MDR phenotype in human MDR1-Pgp expressing cells. Furthermore, exposure of CEM-VBL10 cells to RALT does not induce MDR1-Pgp functional conformation intercepted by monoclonal antibody (mAb) UIC2 binding; nor does exposure to RALT increase the expression of this drug transporter in MDR1-Pgp expressing cells.

**Conclusions:**

No evidence of RALT interaction with human MDR1-Pgp was observed in the *in vitro* MDR cell systems used in the present investigation, this incorporating all sets of studies recommended by the FDA guidelines. Taken in aggregate, these data suggest that RALT may express its curative potential in all sites were HIV-1 penetrates, including the MDR1-Pgp protected blood/tissue barrier. Moreover RALT, evading MDR1-Pgp drug efflux function, would not interfere with pharmacokinetic profiles of co-administered MDR1-Pgp substrate antiretroviral drugs.

## Background

The suboptimal penetration of antiretroviral agents into sanctuary sites such as the central nervous system or into target CD4 cells may contribute to viral persistency. Drug transporters are viewed as one of the major mechanisms which account for suboptimal tissue concentrations of antiretroviral agents. MDR1-P-glycoprotein (MDR1-Pgp, ABCB1), as well as other ABC family members of structurally and functionally related proteins, is a plasma membrane transporter which participates in the transport of a wide variety of drugs, including anti-cancer chemotherapeutics [1] and antiretroviral compounds [2]. The antiviral agent Raltegravir (Isentress®)(RALT) is the first integrase inhibitor (IIN) to be approved for treatment of HIV infection in adults [3,4]. However, the involvement of human drug transporters in RALT absorption, disposition, metabolism and excretion (ADME) has not been fully investigated. RALT has been described as being an MDR1-Pgp substrate [5,6], but there are still few data in the public domain, which are not even definitive.

As for all known anti-retrovirals, the emergence of viral mutations conferring resistance to antiretroviral agents has been documented for this compound [7]. However, drug resistance may also be caused by the biological activity of MDR1-Pgp and/or other members of the ABC transporter family which, through intercepting drugs by means of the binding transport sites within the MDR1-Pgp binding pocket, delivers them out of the cells *via* an ATP dependent mechanism [8,9]. MDR1-Pgp was initially studied in the setting of anticancer treatment; it was identified as the biological entity conferring the multidrug resistance (MDR) in tumor cells, this by reducing the level of cytotoxic drug under sub-lethal concentration [10]. *In vitro* and *in vivo* studies have shown that all protease inhibitors display a high affinity for MDR1-Pgp [11-13], as well the CCR5 inhibitor maraviroc [6,14] and quinolonyl diketoacid derivatives (DKA) with anti-integrase activity [15]. These latter compounds, although different in chemical structure from RALT, exert a similar inhibition on strand transfer activity of HIV-1 integrase. Since *in vivo* efficacy of this class of drugs depends on their access to intracellular sites where HIV-1 replicates, and given that limited information exists on RALT interaction with human MDR1-Pgp expressing cells, we performed a set of well-established *in vitro* studies on the human CD4 positive lymphoblastoid CCRF-CEM cell line and its derivative MDR variants, in line with FDA concept paper on drug interactions [16]. In order to strengthen the data about the interaction between RALT and human MDR1-Pgp, we incorporated an additional human MDR cell system in this investigation. In line with FDA recommendations, we evaluated RALT as substrate, inhibitor and inducer of MDR1-Pgp by performing the following studies: i) inhibition of drug transport function by using the classical efflux assay [17]; ii) down-modulation of multidrug resistance (MDR) phenotype in cell proliferation assay [18]; iii) up-modulation of the monoclonal antibody (mAb) UIC2 epitope in MDR cells during MDR1-Pgp-mediated drug transport [19]; and iv) induction of MDR1-Pgp expression by exposing MDR CEM-VBL10 cells to MDR1-Pgp substrates [20].

## Results and discussion

### Assessment of MDR1-Pgp expression level in human MDR cell lines

**Figure 1:**
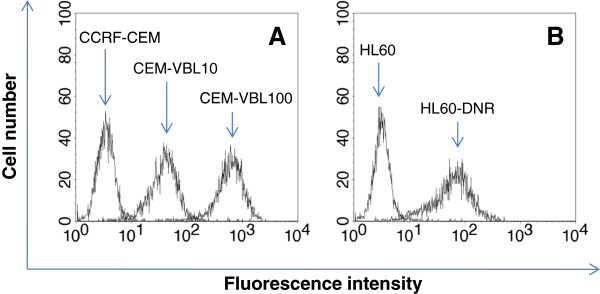
**MDR cell lines.** MDR1-Pgp expression was determined by the highly specific mAb MM4.17. In **Panel A**, the binding profiles obtained by staining the parental drug sensitive cell line CCRF-CEM and its derivative MDR variants (CEM-VBL10 and CEM-VBL100) are shown. In **Panel B**, there are the binding profiles of the AML drug sensitive/resistant cell pairs HL60 and HL60-DNR.

The studies for evaluating the functional and biological interaction of RALT with human MDR1-Pgp were conducted by using two different human cell systems consisting of: a) the lymphoblastoid CD4 positive cell line CCRF-CEM and its derivative MDR variants CEM-VBL10 and CEM-VBL100 expressing increased level of MDR1-Pgp binding sites and relative resistance; b) the drug sensitive/resistant HL60 and HL60-DNR cell pairs of acute myeloid leukemia (AML) origin. The MDR phenotype of such cells was tested and monitored by the highly specific mAb MM4.17 to the external MDR1-Pgp domain [21]. The binding profiles shown in Figure 1 confirm the MDR nature of CEM-VBL10, CEM VBL100 and HL60-DNR cells, while the parental drug sensitive cell lines CCRF-CEM and HL60 were not recognized by the mAb, thereby indicating the absence of detectable MDR1-Pgp molecules.

### Drug efflux

**Figure 2:**
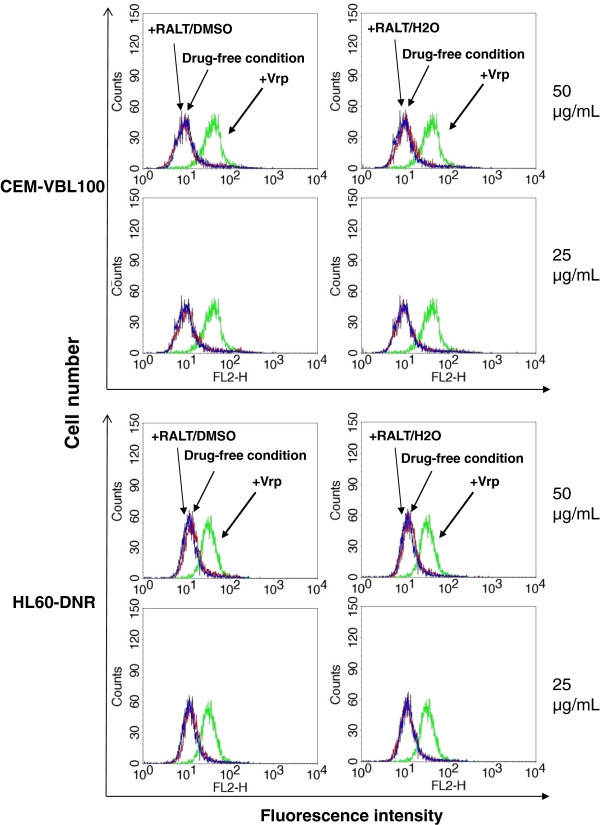
**Evaluation of Rh123 transport inhibition mediated by RALT.** The efflux of the dye MDR1-Pgp substrate Rh123 in CEM-VBL100 and HL60-DNR MDR cells was monitored in drug-free conditions (blue histogram), in the presence of the potent MDR1-Pgp blocker Vrp (2.5 μg/mL) (green histogram), and following incubation with RALT (red histogram) dissolved in DMSO or H2O at the concentrations shown on the right side of the panels.

Rhodamine 123 (Rh123) is a fluorescent marker substrate for MDR1-Pgp; incubation of MDR1-Pgp-positive cells with this drug, followed by washing and further incubation at 37°C, results in a diminished fluorescence profile due to the active drug transport exerted by the MDR1-Pgp efflux system expressed in MDR cells. The presence of a MDR1-Pgp inhibitor such as Verapamil (Vrp) during incubation and/or drug extrusion, restores Rh123 fluorescence [17]. As shown in Figure 2, differently from the potent MDR1-Pgp drug transporter inhibitor Vrp, RALT is not capable at the indicated concentrations to inhibit drug efflux and does not produce Rh123 accumulation in both CEM-VBL100 and HL60-DNR MDR cells. In order to verify the absence of inhibitory effect of RALT on MDR1-Pgp function, additional drug efflux assays were performed with VBL-bodipy and Calcein-AM: these studies showed that the drug does not affect the efflux of the fluorescent dye substrate VBL-bodipy while a little shift involving 5-10% of the Calcein-AM treated cells was observed (Additional file 1: Figure S1). Therefore, these data confirm the absence of a remarkable inhibitory activity of RALT on MDR1-Pgp expressing cells.

### Proliferation assay

**Figure 3:**
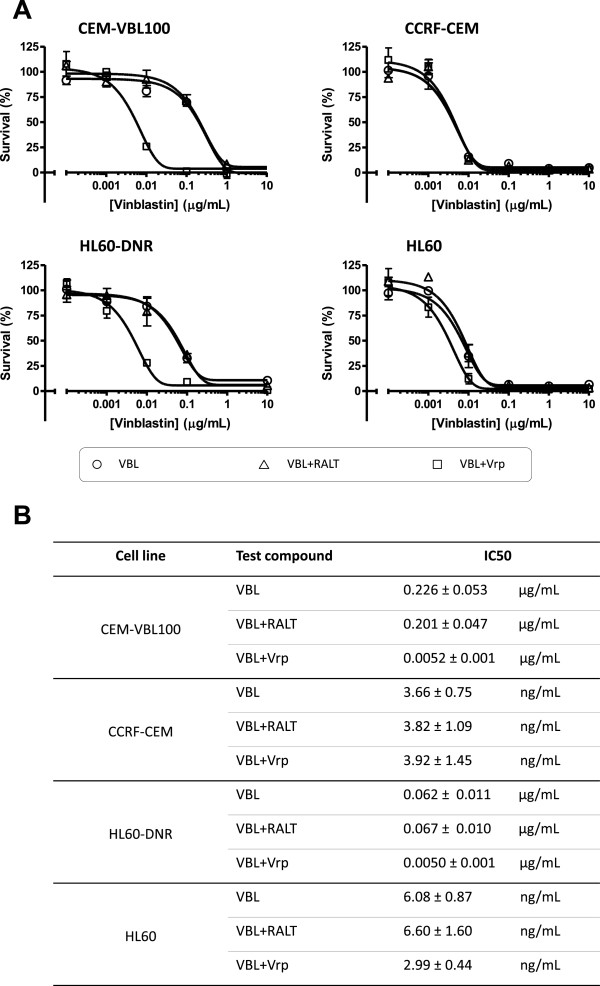
**MDR chemosensitization.** In the upper part of the figure the dose-response growth curves of drug sensitive parental cell lines (CCRF-CEM and HL60) and their derivative MDR variants (CEM-VBL100 and HL60/DNR) are shown **(Panel A)**. The cells were cultured for 72 h in medium containing increasing concentrations of VBL alone (open circles), VBL plus 12.5 μg/mL of the IIN RALT (open triangles), and VBL plus 2.5 μg/mL of the MDR1-Pgp blocker Vrp (open squares). In the lower part of the figure **(Panel B)**, the IC50 values (concentrations of the compound that inhibits cell growth by 50%) for each cell culture condition are reported. Values are means of three independent experiments, each done in triplicate.

**Figure 4:**
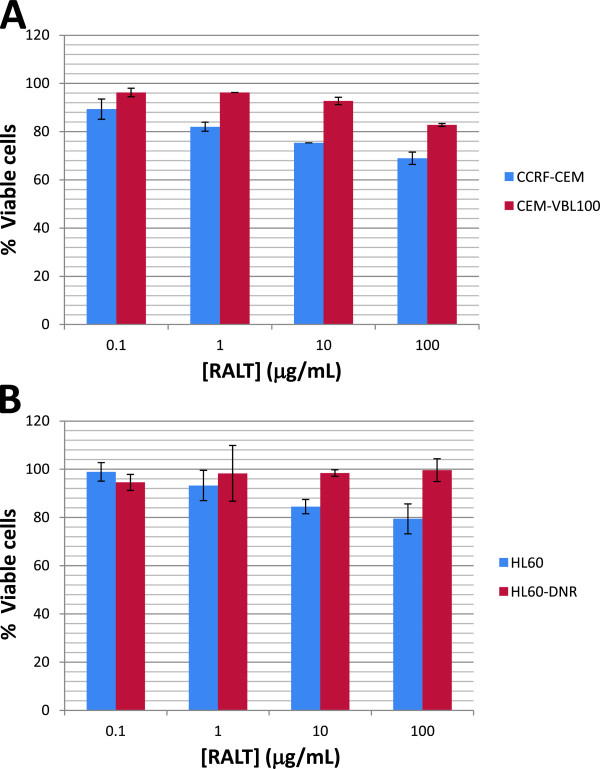
**Growth inhibition assay.** Concentration-dependent effect of the RALT on proliferation rate of drug sensitive/resistant cell pairs CCRF-CEM and HL60 (**Panels A** and **B**, respectively) after 72 h of culture. The figure depicts one representative experiment, and data are expressed as % of untreated control cells with each concentration tested in triplicate.

To evaluate RALT’s potential ability to down modulate the MDR phenotype, standard proliferation assays were used. The experiments were performed by using increasing concentrations of the potent cytotoxic drug vinblastine (VBL) in presence and absence of RALT and the MDR reversing agent Vrp. The concentration of RALT equal to 12.5 μg/mL used in proliferation assays, is twice that observed in plasma of patients treated with conventional RALT dosage [22]. The study was conducted both on drug sensitive parental cells and their derivative variants CEM-VBL100 and HL60-DNR. The growth curves shown in the Figure 3 demonstrate that RALT does not induce any modulation of MDR phenotype on CEM-VBL100 and HL60-DNR cell lines; this suggests that this antiviral drug does not interfere with MDR1-Pgp drug transport function. The cell growth patterns of CEM-VBL100 cells (Figure 3, Panel A) obtained in the presence of VBL and VBL plus RALT are similar as evidenced by their IC50 values (0.226 ± 0.053 μg/mL and 0.201 ± 0.047 μg/mL, respectively) (Figure 3, Panel B). Likewise, the cell growth profiles obtained with HL60-DNR in VBL and VBL plus RALT (Figure 3, Panel A) containing cell culture conditions may be considered comparable in terms of IC50 values (0.062 ± 0.011 μg/mL and 0.067 ± 0.010 μg/mL, respectively) (Figure 3, Panel B). In contrast, the growth curve profiles of both CEM-VBL100 and HL60-DNR MDR cells cultured in the presence of VBL and the MDR reversing agent Vrp, show a dramatic inhibition of cell proliferation caused by down modulation of MDR1-Pgp activity. The IC50 values calculated for the CEM-VBL100 and HL60-DNR in the presence of Vrp were 0.0052 ± 0.0010 μg/mL and 0.0050 ± 0.0010 μg/mL, respectively (Figure 3, Panels A and B). The cell growth curve patterns of the parental drug sensitive CCRF-CEM and HL60 cell lines show, as expected, a higher susceptibility to VBL, while no further biological effect was observed in the presence of the various combinations of drugs (Figure 3, Panels A and B). In order to better elucidate the potential interaction of RALT with MDR1-Pgp, we exposed the panel of cell lines to different amounts of RALT ranging from 0.1 to 100 μg/mL. In this case, the parental drug sensitive cells seem to be more susceptible to RALT in respect to their MDR variants (Figure 4). Considering the small magnitude of growth inhibition, this phenomenon may simply reflect a different drug susceptibility among the cell types observed in *in vitro* conditions. However, it cannot be ruled out that RALT may behave as a weak substrate, and that the MDR1-Pgp molecules expressed on MDR cells act as a drug transporter lowering the drug concentration and its related cytotoxic effect. This result might justify the observation of the small fraction of MDR cell population retaining the dye substrate Calcein-AM in the drug efflux studies (Additional file 1: Figure S1). Furthermore, this hypothesis may be in partial in agreement with previous published findings showing that a reduction of RALT efflux of only 32% was observed when the potent reversing agent Tariquidar was used to inhibit ABCB1 in CEM VBL100 cells [5]. To this regard, it should here mentioned as FDA guidelines recommend that a drug should achieve an efflux ratio greater than a 50% reduction when an ABCB1 inhibitor is used in order for ABCB1 transport to be considered relevant *in vivo*[16]. To further investigate the RALT modulating effect on MDR1-Pgp, the mAb UIC2 shift assay was used. This test is a useful tool for detecting conformational changes associated with the function of MDR1-Pgp and provides a potentially useful diagnostic test for both the expression and the function of MDR1-Pgp [19].

### UIC2 shift assay

**Figure 5:**
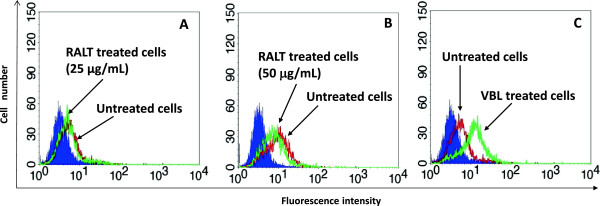
**Modulation of the UIC2 epitope.** In the **Panel A** and **B** the binding profiles of the mAb UIC2 on CEM-VBL10 MDR cells are shown (red histogram); incubation of the cells with RALT (25 and 50 μg/mL) does not interfere with mAb UIC2 binding (green histogram). Conversely, a marked shift of mAb binding UIC2 is observed incubating CEM-VBL10 cells with VBL (10 μg/mL) (**Panel C**, green histogram). The filled profile represents cells stained with secondary antibody alone.

The UIC2 mAb (IgG2a) reacts with the extracellular moiety of MDR1-Pgp and inhibits MDR1-Pgp-mediated efflux of all tested MDR chemotherapeutic drugs. It has been shown that the reactivity of UIC2 mAb on MDR cells is enhanced in the presence of a large array of compounds recognized as MDR1-Pgp substrates which include cytotoxic agents together with certain classes of MDR reversing agents (Verapamil, Quinidine, Cyclosporine-A) [19]. This phenomenon has been used to develop a highly specific and sensitive method to confirm the MDR1-Pgp substrate and MDR reversal agent nature of several chemical agents [23,24]. Therefore, to elucidate the RALT/MDR1-Pgp interaction, we took advantage of the mAb UIC2 ability to bind with increased affinity to its target in the presence of MDR1-Pgp modulators due to conformational changes in functioning MDR1-Pgp. In order to highlight this phenomenon, we used the cell line CEM-VBL10 as MDR1-Pgp expressing cells instead of CEM-VBL100 cells, because of the former’s relatively lower number of MDR1-Pgp binding sites/cell (10,000 binding sites/cell) [M. Cianfriglia, unpublished]. In general, cell lines with a higher level of MDR1-Pgp molecules require higher concentrations of MDR1-Pgp substrates for maximal stimulation [25]. The results of this study show a significant induction of mAb UIC2 binding on CEM-VBL10 cells incubated with VBL (Figure 5, Panel C), while no UIC2 mAb binding shift was observed in presence of 25 and 50 μg/mL of RALT (Figure 5, Panel A and B).

### Induction of MDR1-Pgp expression in MDR cells

**Figure 6:**
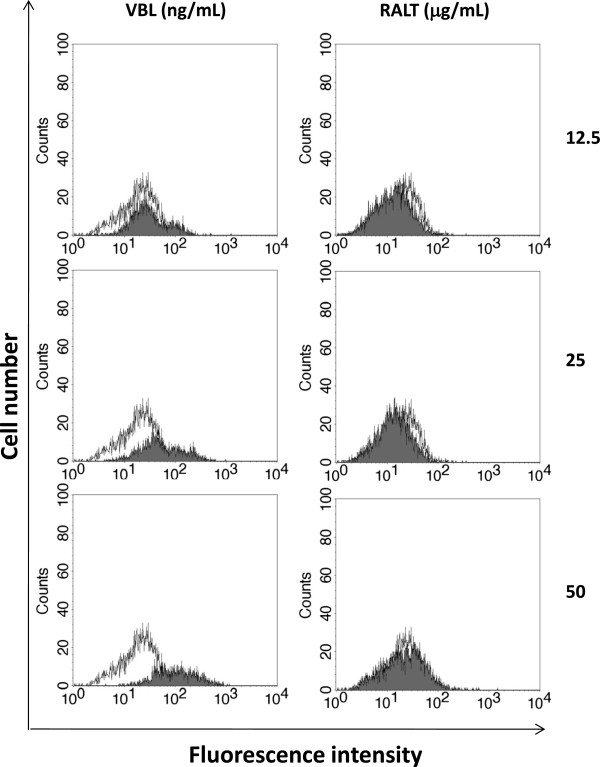
**MDR1-Pgp drug induction assay.** In the left part of the figure, the shift of the MDR1-Pgp binding profile (shaded histogram) is parallel with the increase of VBL concentration. In the right part of the figure, RALT treatment is ineffective in all tested concentrations in up-modulating the MDR1-Pgp expression (empty histogram). In both experiments the highly specific mAb MM4.17 was used for staining procedures.

Since the discovery of the simultaneous resistance of tumor cells to a large array of anti-cancer compounds in the late 1970s, the inhibition of MDR1-Pgp conferring the MDR has become an attractive therapeutic strategy in order to *de novo* sensitize tumor cells to anticancer drugs in cancer patients [26]. However, drug/drug interactions are critical factors in all therapeutic regimens, the co-administration of MDR modulators with drugs that are MDR1-Pgp substrates needing to be balanced with lower drug concentrations to avoid unpredictable side effects [27]. In this regard, the FDA concept paper on drug interactions recommends that new drug candidates should be evaluated as substrates, inhibitors, and also as inducers of MDR1-Pgp [16]. We therefore decided to study the induction of MDR1-Pgp after a prolonged exposure to various concentrations of RALT of CEM-VBL10 cells, which are very prone to modulate MDR1-Pgp expression in the presence of cytotoxic drugs and/or MDR1-Pgp substrates. In parallel experiments, the cells were cultured with increasing amounts of the potent MDR1-Pgp inducer and substrate VBL. The relationship of RALT and VLB concentrations are absolutely empirical, but congruous to demonstrate different induction phenomena exerted by these drugs. In the presence of VBL, CEM-VBL10 cells "respond" by increasing the percentage of MDR cells in relationship with drug concentration, as evidenced by the progressive shift of the fluorescence profiles obtained with the MDR1-Pgp specific antibody MM4.17 (left part of Figure 6). In contrast, RALT is totally ineffective in inducing MDR1-Pgp expression up to the maximum tested concentration of 50 μg/mL (right part of Figure 6). By exposing CEM-VBL10 to an additional increase of drug concentrations (100 ng/mL of VBL and 100 μg/mL of RALT), a large phenomenon of cell death in the VBL containing cultures was observed, but no particular biological effect with regard to the RALT (data not shown).

Several clinical trials have shown a sustained antiretroviral effect and a good tolerability of RALT in naive and treatment-experienced HIV-1 infected patients [28]. Previous investigations have already reported that RALT has a low propensity for involvement in drug–drug interactions [6]. Update studies on pharmacology profile of RALT are described in the recently published review article by Brainard et al. [29], which reports that RALT is not an inhibitor of the major CYP isozymes, including CYP3A4, UGTs, and MDR1-Pgp. Additionally, it has been reported that RALT is not an inducer of CYP3A4 RNA expression or CYP3A4-dependent testosterone 6 beta-hydroxylase activity [14]. In previous studies conducted by our group, a series of diketoacid-containing derivatives (DKA) functioning as inhibitors of HIV-1 integrase have been described as being MDR1-Pgp ABCB1 substrates [15] with strong MDR1-Pgp inhibitory activity. Elvitegravir [4,6], which has a biochemical formulation similar to DKA, shows marked drug interaction with MDR1-Pgp multi-drug transporter and acts as a strong MDR reversing agent [15]. Our study performed with human MDR cell lines clearly shows that the RALT compound does not inhibit MDR1-Pgp mediated drug transport function. The different level of cytotoxic effect exerted by RALT on drug sensitive/resistant cell pairs (Figure 4) and the low shift of a small fraction of MDR cell population incubated in presence of Calcein-AM may be cell type and dye-substrate related, and not sufficient to establish the existence of an authentic interaction with MDR1-Pgp. In this context, Zambruski et al. [6], include elvitegravir, vicriviroc and to a lesser extent RALT in the list of MDR1-Pgp substrates. However, our own findings concerning RALT seem to suggest otherwise, a possible explanation being in the different cell system used and in the interpretation of data. Again in this regard, Moss et al. showed that RALT has minimal interactions with known drug transporters, and that the rate of MDR1-Pgp-mediated transport *in vitro* is so low that the potential for interactions of this entity is expected to be small [5]. The very low rate of RALT transport by MDR1-Pgp expressing cells may explain the absence of major drug interactions with known potent MDR1-Pgp inhibitors. Furthermore, very recently, Tempestilli et al., [30] showed that darunavir, unlike RALT, may modify the expression and functionality of MDR1-Pgp on human lymphocytes. Taken in aggregate, the above mentioned studies are consistent with a previous report where the co-administration of low-dose ritonavir had no major effect on RALT pharmacokinetics, and no dose adjustment was required for patients [31].

Overall, these findings suggest that, in addition to its well known efficacy and safety, RALT may present an advantage in respect to other anti-retrovirals that are MDR1-Pgp substrate. Indeed, RALT’s biological properties may endow it with a higher therapeutic potential against HIV-1 residing in sanctuaries sites pharmacologically protected by MDR1-Pgp expressed on blood tissue barriers. However, in this context, it is important to remember that, despite MDR1-Pgp is the first discovered and probably the most widely studied ABC transporter protein, there are other ABC transporters involved in clinical MDR and in drug absorption and distribution; these include multidrug resistance proteins (MRPs, ABCCs) and breast cancer resistant protein (BCRP, ABCG2) [32,33]. In particular, MRP1, MRP2, MRP4 and BCRP/ABCG2, together with MDR1-Pgp, are present on many barrier sites such as the blood-brain barrier and on many circulating cells such as lymphocytes, and consequently they could contribute to reduce antiretroviral agents in sanctuary or HIV-1 target sites [34].

## Conclusions

Our investigations demonstrate that RALT is ineffective in inhibiting drug efflux and in down-modulating the MDR phenotype of human CEM and HL60 MDR cells to an extent considered relevant *in vivo* by FDA guidelines [16]. In addition, exposure of CEM-VBL10 to RALT does not induce the functional conformation of MDR1-Pgp intercepted by the shift of UIC2 mAb binding. Furthermore, in contrast to other licensed anti-HIV-1 drugs such as the protease inhibitors, RALT has proved to be ineffective in inducing an increase of MDR1-Pgp expression level in MDR cells in culture conditions. The absence of remarkable RALT/MDR1-Pgp interaction may represent a medically relevant property, although at present its impact in the clinical setting is not totally clear. Further studies are warranted to better define i) the mechanisms underlying the profound functional differences of RALT in comparison with other IINs which behave as MDR-Pgp substrates and MDR reversing agents and, ii) the potential involvement of other ABC drug transporters in RALT absorption and disposition.

## Methods

### Chemicals

The RALT was a kind gift of the Merck company (Pomezia, Rome, Italy); Verapamil (Isoptin) was purchased by Abbott (Latina, Italy); Vinblastine (Velbe) by Eli Lilly (Paris, France); Rhodamine-123 was purchased from Sigma (St. Louis, MO). Vinblastine-bodipy (VBL-bodipy) and Calcein acetoxymethlyl ester (Calcein-AM) were purchased from Molecular Probes (Eugene, OH).

### Cell lines

The multidrug resistant (MDR) variants CEM-VBL10 and CEM-VBL100 cells were isolated by stepwise selection of the parental drug sensitive CCRF-CEM (CEM) in the presence of increasing concentrations of VBL [up to the final concentration of 10 and 100 ng/mL, respectively]. Cells were grown under standard conditions for mammalian cells cultured in suspension. The basic medium (BM) for cell culturing consisted of RPMI-1640 supplemented with 10% foetal calf serum (FCS), L-glutamine (2 mM) penicillin (100 U/mL) and streptomycin (100 U/mL). All these components were purchased from Hyclone (Logan, Utah, USA). Identical culture conditions were adopted for the multidrug resistant (MDR) variants HL60-DNR, kindly provided by Dr. Ruoping Tang (Hopitaux de Paris, Paris, France).

### MDR efflux assay

CEM-VBL100 and HL60-DNR cell lines (1 × 10^6^) were loaded with Rh123 (5 μg/mL)(or with VBL-bodipy, 50 ng/mL, or Calcein-AM, 50 ng/mL) in 1 mL of BM in the presence of RALT (concentrations: 50 and 25 μg/mL) or Vrp (2.5 μg/mL) for 1 h at 37°C. The cells were incubated with Rh123 at the indicated concentrations or with drug diluents (DMSO: 0.5%; H2O). At the end of incubation, the cells were washed in serum-free medium and re-suspended in BM in the presence of RALT or Vrp (drug diluents was added in control samples) for a further 1 h at 37°C. Finally, cells were washed twice with ice cold phosphate-buffered saline (PBS)/FACS, and analyzed in a flow cytometer (FACScan, Becton Dickinson, San Josè, CA).

### Cell proliferation assay

The parental drug sensitive and their MDR derivative cell lines in exponential phase of growth were collected, extensively washed with warm RPMI-1640 and seeded (in triplicate) in 96-well microtiter Costar plates (Costar, Rochester, NY) at a density of 5×10^3^ cells/mL.

For MDR chemosensitization studies, the cells were cultured in BM containing increasing concentrations of VBL ranging from 0 to 10 μg/mL; in parallel, MDR cell cultures containing the different concentrations of VBL were grown in the presence of RALT (12.5 μg/mL) dissolved in water. As a control the MDR reversing agent Vrp was used at the concentration of 2.5 μg/mL in an additionally parallel culture. In growth inhibition assays RALT was tested alone at 4 concentrations spread over a range between 0 and 100 μg/mL. For all above described experiments cell survival was determined by WST-1 assay (PreMix WST-1 cell proliferation kit, Vinci Biochem, Firenze, Italy) after 72 h treatment at 37˚C in 5% CO2. The values describing the concentration-response profiles are calculated as % of appropriated control and represent the mean of three independent experiments, each done in triplicate. The GraphPad Prism statistical analysis program was used.

### Monoclonal antibodies and UIC-2 Shift assay

The mAb UIC2 [19] was kindly provided by Dr. E. Mechetner (Chemicon Inc, Temecula, CA). For determination of MDR1-Pgp expression, the mAb MM4.17 recognizing an extracellular MDR1-P-gp epitope on intact/living human MDR cells [21] was used (data not shown). Both UIC2 and MM4.17 mAbs were used in a highly purified form. The UIC2 shift assay was performed under physiological conditions as previously described [19]. CEM-VBL10 cells (1×10^6^) were resuspended in 1 mL of PBS containing 2% FCS and allowed to equilibrate at 37°C in a water bath for 10 min. The RALT was added to samples (final concentration 25 and 50 μg/mL) and incubated for additional 15 min at 37°C with purified UIC2 mAb (final concentration 12.5 μg/mL). VBL (10 μg/mL), a well known UIC2 shifting agent, was used as positive control do detect the conformation of MDR1-Pgp during drug efflux function. Cells were then washed twice in ice-cold PBS containing 2% FCS with 0.01% sodium azide (Shift Stop Buffer, SSB), stained on ice in SSB for additional 15 min with 5 μg/mL of fluorescein-conjugated goat-antimouse antibody (FITC-GAM, Cappel, West Chester, Pa, USA), washed twice with ice cold PBS/FACS and maintained in ice until flow cytometry analysis.

### Induction of MDR1-Pgp expression in MDR cells

For the evaluation of the induction of MDR phenotype, CEM-VBL10 cells in exponential phase of growth were collected, extensively washed with warm RPMI-1640 and resuspended at the concentration of 5 × 10^4^ cells/mL in BM alone, or in the presence of different VBL concentrations (from 100 ng/mL to 12.5 ng /mL) or RALT (from 100 μg/mL to 12.5 μg/mL) and were seeded in 24-wells Costar plates (Costar, Rochester, NY) for 96 h. At the end of the incubation, the cells were harvested, washed with BM alone, and incubated with 12.5 μg/mL of mAb MM4.17. After 30 min of incubation at 4°C, the cells were washed, pelleted, resuspended and incubated for an additional 30 min at 4°C in the presence of fluorescein-conjugated goat antimouse antibody (FITC-GAM, Cappel). After incubation, the cells were washed, resuspended in PBS and processed for flow cytometry analysis.

### Data presentation

All the experiments were repeated at least thrice. The significance was assessed by Student's t-test and the criterion for statistical significance was set at P < 0.05.

## Abbreviations

RALT: Raltegravir; IIN: Integrase inhibitor; MDR1-Pgp and ABCB1: MDR1-P-glycoprotein; MDR: Multidrug resistance; DKA: Quinolonyl diketoacid derivatives; mAb: Monoclonal antibody; Rh123: Rhodamine-123; Vrp: Verapamil; VBL: Vinblastine.

## Competing interests

The authors declare that they have no competing interests.

## Authors’ contributions

CM and LP: conceived and planned the biological approach of this study, participated in the design and coordination of the research and drafted the manuscript. DML and AA: conceived and conducted all the cell biological experiments to demonstrate the biological function of the integrase inhibitor. SV: coordinated and supervised the study. All authors read and approved the final manuscript.

## Pre-publication history

The pre-publication history for this paper can be accessed here:

http://www.biomedcentral.com/2050-6511/14/47/prepub

## Supplementary Material

Additional file 1: Figure S1 Evaluation of VBL-bodipy and Calcein-AM transport inhibition mediated by RALT. The efflux of the fluorescent dyes MDR1-Pgp substrate VBL-bodipy (upper part of the Figure) and Calcein-AM (lower part of the Figure) on CEM-VBL100 MDR cells was monitored in drug-free conditions (red histogram), in the presence of the potent MDR1-Pgp blocker Vrp (2.5 μg/mL) (green histogram), and following incubation with 50 μg/mL RALT (blue histogram) dissolved in DMSO or H2O. Click here for file
