# Supplementary material for: Raltegravir does not revert efflux activity of MDR1-P-glycoprotein in human MDR cells
Source: BMC Pharmacol Toxicol. 2013 Sep 20;14:47. doi: 10.1186/2050-6511-14-47 (PMC3852167; doi:10.1186/2050-6511-14-47)
Supplement: Additional file 1: Figure S1 — Evaluation of VBL-bodipy and Calcein-AM transport inhibition mediated by RALT. The efflux of the fluorescent dyes MDR1-Pgp substrate VBL-bodipy (upper part of the Figure) and Calcein-AM (lower part of the Figure) on CEM-VBL100 MDR cells was monitored in drug-free conditions (red histogram), in the presence of the potent MDR1-Pgp blocker Vrp (2.5 μg/mL) (green histogram), and following incubation with 50 μg/mL RALT (blue histogram) dissolved in DMSO or H2O. [file 2050-6511-14-47-S1.ppt]

## Slide 1
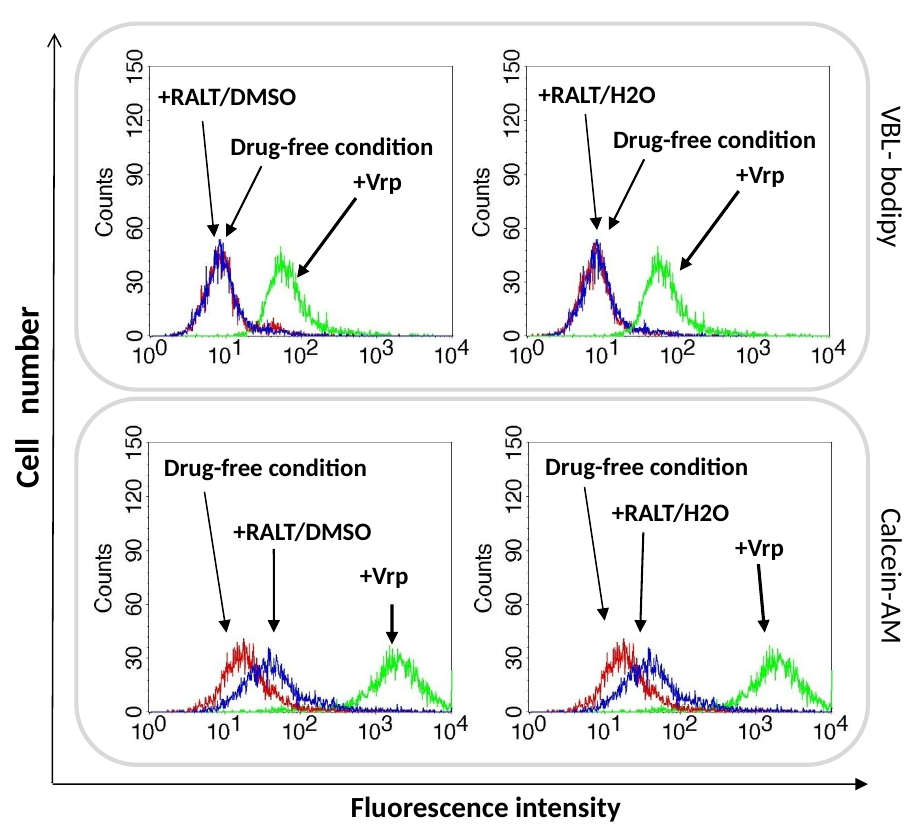

+RALT/H2O
+RALT/DMSO
Drug-free condition
Drug-free condition
+Vrp
+Vrp
VBL- bodipy
Cell number
Drug-free condition
Drug-free condition
+RALT/H2O
+RALT/DMSO
+Vrp
+Vrp
Calcein-AM
Fluorescence intensity
